# Impact of impaired intrinsic capacity on postoperative frailty in elderly patients undergoing colorectal surgery: study protocol for a single-center, prospective, cohort study

**DOI:** 10.3389/fnagi.2025.1523642

**Published:** 2025-05-12

**Authors:** Jirun Wang, Shaoqi Tian, Lei Zhang, Jia Liu, Yuefang Liu, Lei Zhu, Peipei Shan, Ping Zhao, Yang Zhao, Youzhuang Zhu

**Affiliations:** ^1^Department of Anesthesiology, The Affiliated Hospital of Qingdao University, Qingdao, Shandong, China; ^2^Department of Joint Surgery, The Affiliated Hospital of Qingdao University, Qingdao, Shandong, China; ^3^Institute of Translational Medicine, The Affiliated Hospital of Qingdao University, College of Medicine, Qingdao University, Qingdao, Shandong, China; ^4^Department of Anesthesiology, Shengjing Hospital of China Medical University, Shenyang, Liaoning, China

**Keywords:** frailty, intrinsic capacity, colorectal surgery, elderly patients, cohort study

## Abstract

**Introduction:**

Frailty is characterized by a decline in multiple physiological systems, increasing vulnerability to stressors such as surgery and anesthesia. A decline in intrinsic capacity is common among elderly populations and has been demonstrated to be a predictor of frailty in community-dwelling seniors. However, the relationship between preoperative intrinsic capacity decline and postoperative frailty in surgical patients remains unclear.

**Methods:**

This study is a single-center, prospective, cohort study. The study will recruit participants aged 60 years and above who are scheduled to undergo elective colorectal surgery. Participants will be classified into an exposed group (intrinsic capacity score ≤ 8) and a non-exposed group (intrinsic capacity score ≥ 9) according to their preoperative intrinsic capacity assessment. The primary outcome is the risk of frailty in elderly patients with impaired intrinsic capacity within one year following colorectal surgery. The secondary outcomes include postoperative pain scores, sleep quality, recovery quality, grip strength, fall risk, activities of daily living, onset time of moderately frailty, incidence of moderately frailty, and adverse events. All assessments will be conducted at predetermined intervals through face-to-face interviews during hospitalization and via telephone follow-up post-discharge.

**Discussion:**

This study aims to clarify the risk of postoperative frailty in older patients with impaired intrinsic capacity. This study seeks to enable the early identification of patients with impaired intrinsic capacity, allowing for the implementation of targeted interventions to reduce the risk of postoperative frailty.

## 1 Introduction

### 1.1 Background and rationale

#### 1.1.1 Frailty

The global population is undergoing rapid aging. According to data from the World Health Organization (WHO), individuals aged 60 and above accounted for 12% of the world’s population in 2015, with projections indicating an increase to 22% by 2050 (Beard et al., 2016). With advancing age, various health issues become increasingly prevalent, with frailty standing out as a critical concern. Frailty is defined as a syndrome marked by the deterioration of multiple physiological systems, leading to reduced resilience against stressors (Hoogendijk et al., 2019). Frailty commonly presents with decreased skeletal muscle strength, weight loss, fatigue, cognitive decline, and recurrent infections. Primarily affecting older adults, the preoperative incidence of frailty in elderly surgical patients ranges from 18.6% to 56% (Eide et al., 2015; Jung et al., 2015; Khan et al., 2016; Itagaki et al., 2020; Mahanna-Gabrielli et al., 2020) Frailty is associated with heightened vulnerability to adverse health outcomes, including increased mortality, likelihood of institutionalization, reduced quality of life, higher readmission rates, and extended hospital stays (Arya et al., 2015; Brahmbhatt et al., 2016; Fagard et al., 2016; Persico et al., 2018; Watt et al., 2018).

#### 1.1.2 Mechanisms and vulnerability factors in frailty

The biological mechanisms underlying frailty remain inadequately elucidated. Potential mechanisms include aging, chronic inflammation, mitochondrial dysfunction, cellular senescence, deregulated nutrient sensing, calcium homeostasis imbalance, oxidative stress abnormalities, cytoskeleton damage, hormonal dysregulation, reactive oxygen metabolism obstruction, epigenetic changes, or immune responses (Cardoso et al., 2018; Álvarez-Satta et al., 2020; Kim and Rockwood, 2024). Numerous studies have identified a variety of vulnerability factors that significantly contribute to frailty. These factors include nutritional influences, behavioral aspects (such as smoking, sedentary lifestyle, poor sleep quality, and prolonged bed rest) (Marissa et al., 2016), social determinants (including low educational attainment and socioeconomic status) (Young et al., 2016), environmental influences, genetic predispositions (Mak et al., 2022), and the presence of comorbidities (Marissa et al., 2016). Under natural conditions, the reversal of frailty presents significant challenges, with only approximately 25% of patients transitioning from a frail state to a pre-frail state. Furthermore, elderly individuals with comorbidities, such as diabetes and stroke, are at a heightened risk of experiencing an exacerbation of their frailty (Lee et al., 2014). Current interventions aimed at mitigating frailty frequently concentrate on addressing these vulnerability factors. These interventions explore their effects on the onset and progression of frailty and include strategies such as fall prevention training (Apóstolo et al., 2019; Chittrakul et al., 2020), enhancement of gut microbiota (Ghosh et al., 2020), diabetes management (Simpson et al., 2023), perioperative exercise (Waite et al., 2017; Apóstolo et al., 2019), pulmonary function training (Maddocks et al., 2016), and cognitive function training (Apóstolo et al., 2019).

#### 1.1.3 Intrinsic capacity and frailty

Intrinsic capacity (IC) is characterized by the composite of physical and mental capacities that an individual can employ (World Health Organization [WHO], 2015). IC encompasses five essential domains: locomotion, cognition, vitality, sensory, and psychological. The five domains are interconnected and inseparable, and damage to any one domain may directly or indirectly affect the others. A decline in IC can lead to negative consequences, including functional limitations, social isolation, increased frailty, disability, increased socioeconomic burdens, and heightened mortality (González-Bautista et al., 2021; Beard et al., 2022). Both frailty and IC decline are conceptual frameworks utilized to characterize the physical aging process in elderly patients. IC and frailty can be seen as two sides of the same coin with many fundamental differences in their underlying logic (Liu et al., 2021b). The most comprehensive and accurate assessment tool for frailty is the Frailty Index (FI) proposed by Rockwood and Mitnitski (2007), which describes the physical aging process by emphasizing the accumulation of health deficits as an overall state. In contrast, the concept of intrinsic capacity (IC) focuses more on an individual’s residual biological capacity and adaptability (Cesari et al., 2019; Zhou and Ma, 2022; Hamaker et al., 2023), resembling a phenotype approach. The FI was developed based on elderly individuals in Canada. A significant proportion of the participants are nursing home residents who are highly dependent on others for daily living and have severe functional impairments (Rockwood and Mitnitski, 2007). In comparison, the ICOPE assessment tool has a broader applicability and has been validated in various community-dwelling populations (Rodríguez-Laso et al., 2023; Ma et al., 2024; Zhou et al., 2024). However, these two concepts are not mutually exclusive. Belloni and Cesari (2019) argue that IC can be seen, to some extent, as an evolution of the concept of frailty, and that these two concepts are complementary. Monitoring IC helps to predict the onset and development of frailty. Evaluating IC in populations at elevated risk for frailty is crucial, as it facilitates the formulation of personalized care interventions tailored to individual preferences and specific health conditions. Gutiérrez-Robledo et al. (2021) argue that the IC serves as a critical determinant in differentiating between frailty, pre-frailty, and robust states in adults, demonstrating a significant correlation between the IC index and frailty. Therefore, further research is needed to unravel the complex relationship between IC and frailty.

In the Chinese population, the prevalence of IC decline is approximately five times higher than that of frailty (Ma et al., 2021). Declines in IC may occur even in individuals who are healthy or in a pre-frail state (Liu et al., 2021a). Assessing IC helps identify high-risk individuals early, thereby facilitating timely interventions to prevent the onset of frailty. Research has established that a decline in IC is a significant predictor of frailty in both community-dwelling populations and hospitalized patients (Chew et al., 2021; Shen et al., 2023). However, the relationship between a decline in IC and the risk of postoperative frailty remains to be fully elucidated.

It is well recognized that surgery and anesthesia impose significant physiological stress on patients (Desborough, 2000). For those with frailty, an elevated risk for a range of complications including atelectasis, pneumonia, myocardial injury, acute kidney injury, and postoperative delirium is undeniable (Ferraris et al., 2014; Rothenberg et al., 2019). But for those who have not yet attained a state of frailty but exhibit diminished IC, the stress associated with surgical and anesthetic procedures may not only lead to complications, but also compromise their resilience, thereby potentially accelerating their transition to a frail state.

### 1.2 Objectives

The objective of this study is to evaluate the risk of frailty in elderly patients with compromised IC within one year following colorectal surgery. The hypothesis of this study posits that the risk of developing frailty within one year following colorectal surgery is elevated in the exposed group relative to the non-exposed group.

## 2 Materials and methods

### 2.1 Study design and study setting

**FIGURE 1:**
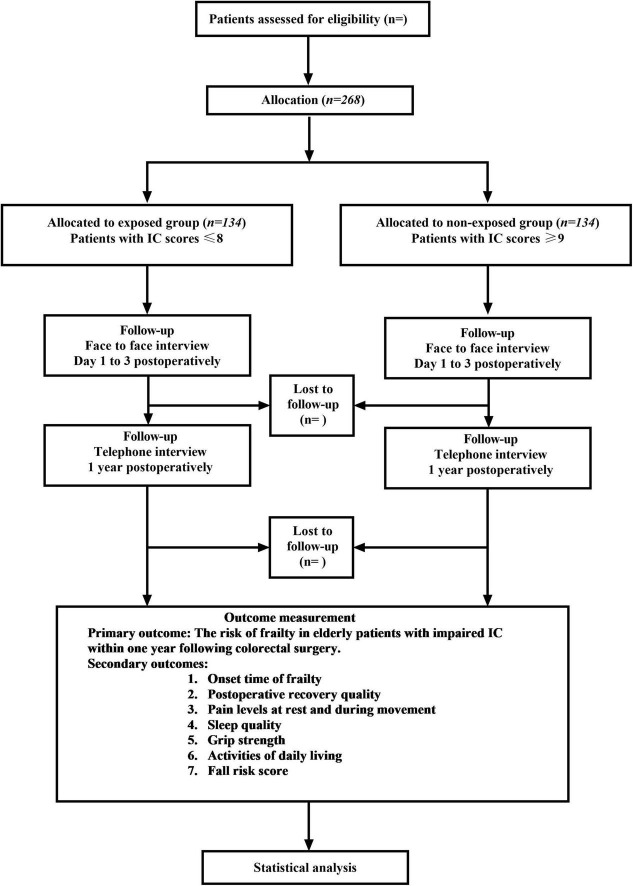
Participants exposure, outcome measures, and follow-up.

This study is a single-center, prospective, cohort study that is scheduled to take place at The Affiliated Hospital of Qingdao University. Participants will be assigned to exposed and non-exposed groups based on intrinsic capacity scores (Figure 1). Data collection is scheduled to occur from July 2024 to December 2026. A long-term follow-up will be conducted for each eligible patient, extending from the time of admission through one-year post-discharge. This study will adhere to the established protocol guidelines outlined in the Strengthening the Reporting of Observational Studies in Epidemiology (STROBE) statement. Prior to trial initiation, written informed consent will be obtained from each patient capable of providing consent. In cases where the patient lacks decisional capacity, as determined by a cognitive assessment (Mini-Mental State Examination < 20) (Sessums et al., 2011), consent will be obtained from a legally authorized representative (proxy) in accordance with ethical guidelines.

### 2.2 Eligibility criteria

#### 2.2.1 Inclusion criteria

The study population comprises individuals aged 60 years and older who were scheduled to undergo colorectal surgery under general anesthesia. The exposed group includes patients with impaired IC, defined as an IC score of 8 points or lower. Conversely, the non-exposed group consists of patients with robust IC, characterized by an IC score of 9 points or higher, with the maximum possible score being 10 points.

#### 2.2.2 Exclusion criteria

(i) Patients exhibiting preoperative frailty. (ii) Patients undergoing emergency surgical procedures. (iii) Individuals presenting with acute illnesses, such as acute heart failure, acute coronary syndrome, acute infection, organ dysfunction, acute psychiatric conditions, acute exacerbation of chronic obstructive pulmonary disease, and acute pneumonia. (iv) Patients with impaired consciousness resulting from severe neurological or psychiatric disease, or those unable to communicate effectively due to low educational attainment or language barriers. (v) Individuals with alcohol or substance use disorders or dependence. (vi) Participants who have been involved in other clinical studies within the past year.

### 2.3 Exposure

Researchers will evaluate the IC of patients one day before surgery. This comprehensive assessment will be grounded in the five dimensions of IC as defined by the WHO: locomotion, cognition, vitality, sensory, and psychological. The following methodologies will be employed to assess IC in this study: Cognitive capacity will be evaluated through measures of time orientation, memory, and processing (Cai et al., 2012); Locomotion will be assessed via walking speed, the sit-to-stand test, and balance (Guralnik et al., 1994); Vitality will be gauged through nutritional status (Kaiser et al., 2009); Sensory capacity will be examined through hearing and vision tests (Liu et al., 2023); and Psychological will be assessed through measures of depression (Lim et al., 2000).

#### 2.3.1 Cognition

Cognitive function is evaluated using the Mini-Mental State Examination (MMSE), a 30-item assessment tool designed to measure multiple dimensions of cognitive performance. These dimensions include immediate memory, temporal and spatial orientation, delayed recall, language proficiency, visuospatial skills, attention, and calculation abilities. The MMSE yields a maximum score of 30, with higher scores denoting superior cognitive function (Cai et al., 2012). Scores are categorized into three distinct ranges: severe cognitive impairment (0–9), mild to moderate cognitive impairment (10–26), and normal cognitive function (27–30).

#### 2.3.2 Locomotion

Locomotion is assessed using the Short Physical Performance Battery (Guralnik et al., 1994), which includes three components: standing balance, chair stands, and walking speed tests. Each component is scored on a scale from 0 to 4, with a maximum total score of 12. For the balance assessment, we assess whether patients can maintain three different standing positions for 10 s each: feet together, semi-tandem, and tandem. A score of “1” will be given if the participant can maintain feet together but not the semi-tandem standing position for 10 s. A score of “2” will be given if the participant can maintain the semi-tandem standing position for 10 s but not the tandem standing position for more than 3 s. A score of “3” will be given if the participant can maintain the semi-tandem standing position for more than 10 s and the tandem standing position for 3–9 s. A score of “4” will be given if the participant can maintain the tandem standing position for 10 s. For the walking test, we assess the patient’s walking speed for 4 meters on flat ground, repeating the test twice and selecting the shortest time as the test result. Scores are categorized based on the time taken: 1 point for over 8.70 s, 2 points for 6.21–8.70 s, 3 points for 4.82–6.20 s, and 4 points for under 4.82 s. For the chair standing test, there are two tests consisting of a single standing and five standings. For the single standing, patients cross their arms and stand up once from a seated position. Scores are assigned as follows: 0 points if the single standing is not completed or if the time for five standing exceeds 60 s, 1 point for a time between 16.70 and 60 s for five standing, 2 points for a time between 13.70 and 16.69 s, 3 points for a time between 11.20 and 13.69 s, and 4 points for a time under 11.19 s. After completing these assessments, the scores from all three components are summed. A total score of ≤ 2 indicates moderate to severe impairment in locomotion, 3–9 points indicate mild to moderate impairment, and 10–12 points indicate normal locomotion. In this study, a score of ≤ 9 points indicates a decline in the locomotion domain.

#### 2.3.3 Vitality

In the vitality domain, we will measure by using the Mini Nutritional Assessment Short Form, a 6-item screening questionnaire validated as a sensitive tool for rapid nutritional screening. The highest score is 14 points, with scores less than 8 indicating malnutrition and scores between 8 and 11 indicating a risk of malnutrition (Kaiser et al., 2009).

#### 2.3.4 Sensation

The sensory domain primarily includes auditory and visual functions, assessed through self-report methods. Visual function is primarily assessed by asking elderly patients, “How do you feel about seeing things at a distance? For example, can you recognize a friend across the street (with or without glasses)?” Hearing function is assessed by asking elderly patients, “How is your hearing?” (Liu et al., 2023). In this study, impairment in either hearing or vision scores 1 point, while impairment in both scores 0 point.

#### 2.3.5 Psychological

In the psychological domain, assessment is conducted using the 15-item Geriatric Depression Scale (GDS-15) (Lim et al., 2000), which consists of 15 questions with a total score of 15 points. Mild depression is defined as a GDS-15 score ≥ 5, while severe depression is defined as a score ≥ 10.

#### 2.3.6 A composite score for intrinsic capacity

López-Ortiz et al. (2022) proposed a composite score for IC, integrating cognitive, locomotion, vitality, sensory, and psychological dimensions into a single measure. Each dimension is assessed on a scale ranging from 0 to 2, with 0 indicating severe impairment, 1 indicating partial impairment, and 2 indicating minimal impairment or normal function. The total IC score ranges from 0 (worst) to 10 (best). The present study employs a consistent calculation method wherein the total IC score is derived by summing the individual scores for locomotion, cognition, vitality, sensory, and psychological. The scores from all dimensions are summed to form a complete IC score, where 0–4 indicates significant loss of capacity, 5–8 indicates declining capacity, and 9–10 indicates high and stable capacity. Lower scores indicate a more pronounced decline in IC, and ≤ 8 points denote IC decline in this study.

### 2.4 Outcomes

#### 2.4.1 Primary outcome

The risk of frailty in elderly patients with impaired IC within one year following colorectal surgery. In this study, Clinical Frailty Scale (CFS) level 4 or above is classified as frailty, while levels below 4 are classified as non-frailty.

#### 2.4.2 Secondary outcomes

(i) The onset time of frailty (CFS level ≥ 4); (ii) Postoperative recovery quality; (iii) Pain levels at rest and during movement; (iv) Sleep quality; (v) Grip strength; (vi) Activities of daily living; (vii) Fall risk score.

#### 2.4.3 Safety outcomes

Assessment of postoperative adverse events from the time of admission to one year after surgery, including: (i) Reoperation events; (ii) Major adverse cardiovascular and cerebrovascular events, including myocardial infarction, heart failure, malignant arrhythmias, and stroke; (iii) Postoperative pulmonary complications, including pneumonia, respiratory failure, atelectasis, pleural effusion, and pulmonary embolism; (iv) Delirium; (v) Hepatic and renal insufficiencies; (vi) Urinary tract infections; (vii) Falls; (viii) Nausea and vomiting; (ix) Surgery-related complications including anastomotic leakage, hemorrhage, and postoperative infection; (x) Readmission; (xi) All-cause mortality.

### 2.5 Confounders

Studies have demonstrated that confounders affecting frailty and IC include age, sex, BMI, tobacco use, alcohol consumption, socioeconomic status (Feng et al., 2017; Huang et al., 2023), marital status (Kojima et al., 2020), and comorbid conditions such as renal failure, cognitive decline, history of cancer, chronic lung disease, stroke, diabetes, osteoarthritis (Assar et al., 2019; Huang et al., 2023; Wang et al., 2023), medication usage (Pazan et al., 2021; Ryu et al., 2023), laboratory parameters (e.g., hemoglobin, albumin, C-reactive protein level) (Mailliez et al., 2020).

### 2.6 Patient pathway

The surgery is performed by the Gastrointestinal Surgery team at the West Coast Campus of Qingdao University Affiliated Hospital. As a regional medical center, it performs 1,700 colorectal surgeries annually, with an average waiting time of 4 days from admission to surgery and an average hospital stay of 11 days per patient.

All patients scheduled for colorectal surgery under general anesthesia must undergo a standardized preoperative evaluation. This process is carried out by a multidisciplinary team (MDT) consisting of surgeons, anesthesiologists, nursing staff, and, when necessary, specialist physicians to ensure optimal perioperative management.

After admission, the surgeon initially conducts a comprehensive physical examination, confirms the lesion through imaging and pathological tests, and determines the surgical plan. Subsequently, the surgeon and nursing team collaboratively collect the patient’s basic medical history—including previous medical conditions, surgical history, medication usage, lifestyle, and social support—while completing preoperative laboratory and imaging studies to assess the patient’s overall health. These examinations include, but are not limited to, cardiopulmonary evaluations (electrocardiogram, pulmonary function tests, echocardiography, etc.) and laboratory tests (complete blood count, biochemical markers, coagulation profile, infection markers, etc.).

One day before the surgery, the anesthesia team intervenes to conduct a systematic pre-anesthetic evaluation, collecting baseline physiological data and using clinical scales to assess the patient’s frailty and intrinsic capacity, thereby determining their anesthetic tolerance. Based on these results, a personalized anesthesia plan is formulated, and for patients at potential risk, preoperative optimization measures—such as correcting anemia, optimizing blood sugar control, improving nutritional status, and adjusting chronic disease management—are implemented to enhance intraoperative and postoperative safety.

For patients with multiple comorbidities or poorer baseline conditions, the MDT further collaborates with experts from cardiology, neurology, endocrinology, pulmonology, and other relevant specialties to conduct a multidisciplinary evaluation. An individualized perioperative management plan is then developed to optimize the patient’s preoperative condition, reduce surgical risks, and ensure that the surgery is completed safely, thereby accelerating recovery.

The postoperative rehabilitation plan adheres to the Enhanced Recovery After Surgery (ERAS) principles, aiming to promote the recovery of intestinal function, reduce postoperative complications, and speed up overall patient recovery. It includes:

(i) Early mobilization and functional exercises: On postoperative day 1, with the assistance of medical staff, patients are encouraged to sit up, stand, and gradually increase their walking distance. The goal is to achieve 3–5 walking sessions per day, with patients returning as close as possible to their normal daily activity levels within 3–5 days after surgery.
(ii) Breathing exercises and pulmonary rehabilitation: Patients use an incentive spirometer to perform multiple deep breathing exercises daily to prevent atelectasis. They are also encouraged to cough actively to clear secretions, and if necessary, nebulization treatment or physical vibration techniques are employed to facilitate sputum clearance.
(iii) Abdominal core stabilization training: In the early postoperative period, patients perform gentle diaphragmatic breathing exercises and contract the transversus abdominis (core muscles) to strengthen their abdominal muscles. As recovery progresses, exercises such as pelvic tilting (anterior and posterior) and standing knee lifts are gradually introduced to improve abdominal control.
(iv) Pelvic floor muscle rehabilitation: Kegel exercises are implemented to strengthen the pelvic floor muscles, thereby enhancing postoperative bowel function and reducing the risk of urinary incontinence.
(v) Training to improve urinary function: On postoperative days 1–2, medical staff guide patients to intermittently clamp the urinary catheter—typically for 1–2 h at a time—to allow the bladder to gradually adapt to urine storage and to train the detrusor muscle to contract effectively.

### 2.7 Data collection

Investigators involved in data collection will receive rigorous systematic training and will be unaware of patient exposure or overall enrollment status. During the hospitalization period, data will be collected utilizing electronic medical record systems and face-to-face interviews. Following discharge, data collection will be conducted through telephone follow-ups at predetermined intervals, employing standardized instruments (Table 1).

**TABLE 1:** Enrollment and assessments schedules.

Outcome measure		Visit 1	Visit 2	Visit 2	Visit 3	Visit 4	Visit 5
Time		1 day pre-surgery	During operation	POD 1	POD 2	POD 3	Monthly for one -year post-surgery
Enrollment	Eligibility screening	X					
	Informed consent	X					
	Demographic characteristics	X					
	Baseline measures	X	X				
	Allocation	X					
Assessments	Frailty Scale	X					
	Clinical Frailty Scale						X
	Grip strength	X		X	X	X	
	NRS at resting and movement	X		X	X	X	X
	Sleep quality scores	X		X	X	X	X
	Barthel Index			X	X	X	X
	QoR-15 scores			X	X	X	
	Walking ability			X	X	X	X
	Comfort level			X	X	X	X
	Morse Fall Scale			X	X	X	X
	Safety outcomes	Safety outcomes will be assessed from admission through one-year post-surgery					

POD, postoperative day; QoR-15: Quality of Recovery-15; NRS: Numerical Rating Scale.

#### 2.7.1 Baseline data

Demographic characteristics of patients will be assessed the day before surgery, including age, sex, height, weight, marital status (classified as married, single, widowed, and divorced), education level (classified as low [elementary school or less], medium [lower vocational or general intermediate education], and high [intermediate vocational education, general secondary school, higher vocational education, college, or university]), living situation (classified as living alone, living with a spouse, living with family, living with spouse and family), smoking history (classified as regular smokers [engaging in the consumption of more than one cigarette per day for a duration of six months, whether continuously or cumulatively], occasional smokers [smoking more than four times per week, with an average consumption of less than one cigarette per day], never smokers, former smokers [stop smoking for at least two years]), alcohol history (males consuming 500 ml of beer or 50 ml of spirits daily; females consuming 400 ml of beer or 40 ml of spirits daily), the American Society of Anesthesiologists (ASA) classification, comorbidities, medication usage (such as beta-blockers, corticosteroids, and statins), and laboratory data (such as hemoglobin, albumin, C-reactive protein, white blood cell count, neutrophil count, and platelet count). Intraoperative data will be assessed, including type and duration of surgery, type and duration of anesthesia, blood transfusion volume, infusion volume, blood loss and pathological findings (TNM staging). Postoperative conditions will be documented, with particular attention to the administration of thermal infusion therapy and chemotherapy.

#### 2.7.2 Frailty assessment

##### 2.7.2.1 Preoperative frailty screening

Frailty screening will be conducted using the Frail Scale one day before surgery. The scale consists of five questions:

(i) Do you feel tired at least 3 or 4 days per week?
(ii) Can you climb one floor without assistance?
(iii) Can you walk one block or 100 m without assistance?
(iv) Do you suffer from more than five diseases?
(v) Has your weight decreased by ≥ 4.5 kg or 5% of baseline in the previous 12 months?

Patients meeting 3–5 components were classified as frail. Patients presenting with preoperative frailty will be excluded from the study.

##### 2.7.2.2 Postoperative frailty assessment

One-year follow-ups will use the Clinical Frailty Scale (CFS) to assess frailty. The CFS score is a simplified version of the FI proposed by Rockwood et al. (2005), and it still falls within the deficit accumulation model framework. Rockwood and Theou (2020) emphasized that the CFS can serve as an alternative to the FI for rapid clinical assessment of frailty.

**FIGURE 2:**
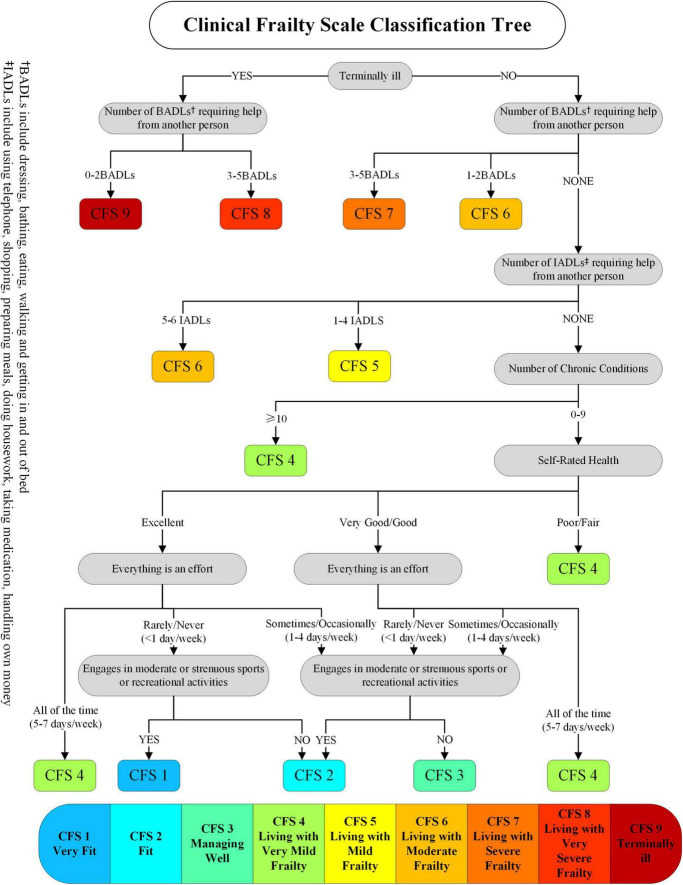
CFS classification tree.

The CFS ranges from 1 to 9, higher values indicate greater frailty (1=̃ very fit, 2=̃ well, 3=̃ managing well, 4=̃ very mildly frail, 5=̃ mildly frail, 6=̃ moderately frail, 7=̃ severely frail, 8=̃ very severely frail and 9=̃ terminally ill). Category 9 is designated for individuals who are terminally ill. The evaluation process will adhere to the criteria outlined in the Clinical Frailty Scale (CFS) classification tree (Figure 2). The classification tree was developed alongside the CFS to assist less experienced raters in scoring the CFS and has shown reasonable reliability, with full agreement in 63% of cases and ± 1 level agreement in an additional 30% of cases when compared to assessments by experienced geriatricians (Theou et al., 2021).

#### 2.7.3 Other outcomes

##### 2.7.3.1 Short-term outcomes will be assessed on postoperative days 1, 2, and 3

(i) The Quality of Recovery-15 (QoR-15) scale is utilized to assess postoperative recovery quality. The QoR-15 questionnaire comprises 15 items that evaluate overall postoperative recovery across five domains: physical comfort (5 items), emotional state (4 items), physical independence (2 items), psychological support (2 items), and pain (2 items). Each item is rated on a scale from 0 to 10, where 0 signifies the absence of the symptom and 10 denotes its persistent or severe presence, with negative indicators scored inversely. Each item is rated on a scale from 0 to 10, with the total score representing the sum of the 15 items, yielding a range from 0 to 150. A higher score on the QoR-15 scale signifies an enhanced quality of postoperative recovery.
(ii) Grip strength was assessed using a hand dynamometer to determine the maximum grip strength of the dominant hand through isometric contraction. Participants were seated on a chair with a straight back and no armrests, ensuring relaxed shoulders and a 90-degree elbow flexion without arm support. Each participant exerted maximum effort during each trial, with three measurements recorded and averaged. A rest period of 5–10 s was allowed between each measurement.

##### 2.7.3.2 Long-term outcomes will be assessed on postoperative days 1, 2, and 3, followed by monthly evaluations for one year

(i) Pain levels are evaluated at rest and during movement using the Numerical Rating Scale (NRS). Rest is operationally defined as lying quietly in a supine position, while movement is characterized by walking on flat ground. The NRS quantifies pain intensity on a scale ranging from 0 to 10, where a score of 0 signifies the absence of pain, scores of 1–3 indicate mild pain, scores of 4–6 represent moderate pain, scores of 7–9 indicate severe pain and a score of 10 reflects unbearable pain.
(ii) The Fall Risk Score is assessed utilizing the Morse Fall Scale, which yields a total score ranging from 0 to 125. Higher scores on this scale are indicative of an increased risk of falling. Specifically, a score exceeding 45 denotes a high risk, scores between 25 and 45 signify a moderate risk, and scores below 25 represent a low risk.
(iii) Activities of Daily Living are evaluated utilizing the Barthel Index. The total score on this index ranges from 0 to 100, with higher scores denoting greater independence and reduced dependence.
(iv) Sleep Quality is assessed utilizing the NRS, which ranges from 0 to 10, with a score of 0 representing excellent or good sleep quality and a score of 10 indicating an inability to sleep throughout the night. Postoperative sleep disturbances are defined as a total NRS score of 6 or higher.

### 2.8 Data Management

The datasets utilized in this study will undergo anonymization and will be securely stored within the Data Management Platform of The Affiliated Hospital of Qingdao University.^1^ Data entry will be conducted by trained personnel adhering to standardized operating procedures. Each participant will be assigned a unique study identifier to maintain anonymity and ensure precise tracking throughout the study. Data coding will conform to predefined coding dictionaries to ensure consistency. Categorical variables will be numerically coded, while continuous variables will be entered as documented in the source materials. All electronic data will be securely stored on password-protected servers with encryption to safeguard against unauthorized access. Access to the data will be restricted to authorized study personnel, with access levels tiered according to roles and responsibilities. The Electronic Data Capture system will utilize audit trails to document any modifications made to the data. Physical data will be maintained in a locked cabinet within a secure location, accessible exclusively to authorized personnel.

### 2.9 Sample size

The existing literature is deficient in studies examining the prevalence of frailty in hospitalized patients following surgical procedures. A cohort study on hospitalized non-surgical patients has shown that the prevalence of frailty is approximately 65.4% (Shen et al., 2023). In this study, the non-exposed group consists of individuals with robust intrinsic capacity, with a follow-up period of one year. Therefore, it is hypothesized that the prevalence of frailty one-year post-surgery will be 50%. Previous study indicates that a decline in intrinsic capacity promotes the occurrence of frailty, with an odds ratio of 2.2 (Shen et al., 2023). At a significance level (α) of 0.05 and a power (1-β) of 80%, the required sample size per group is calculated to be 107 participants using Power Analysis and Sample Size version 15.0 (Stata Corp. LP, College Station, TX, United States). Accounting for a projected dropout rate of 20%, a total of 134 participants per group is necessary, resulting in an overall sample size of 268 participants.

### 2.10 Statistical analysis

This study will use Statistical Package for Social Science (SPSS) version 25.0 and R version 4.3.1 for data analysis. Continuous data will be reported as mean ± standard deviation, or median and interquartile range, whereas categorical data will be presented as rates and percentages. For baseline data, comparisons of continuous variables between the exposed and non-exposed groups will be performed using independent samples *t*-tests or Mann–Whitney U tests. Comparisons of categorical variables will be performed using chi-square tests, continuity-corrected chi-square tests, or Fisher’s exact tests. The primary outcome is the risk of frailty, and the relative risk (RR) and 95% confidence interval (CI) will be calculated using a modified Poisson regression model. We will construct three models to assess the association between impaired intrinsic capacity and postoperative frailty. Model 1 will only include exposure factors without adjusting for other covariates. Model 2 will adjust for covariates including age, sex, height, weight, marital status, education level, living status, smoking history, drinking history, ASA classification, comorbidities, preoperative medication, and laboratory tests. Model 3 will further adjust for intraoperative variables, including type and duration of surgery, type and duration of anesthesia, volume of blood transfusion, infusion volume, and blood loss.

For the secondary outcome analysis, propensity score matching will be employed. Intrinsic capacity will be used as the dependent variable, and baseline variables with imbalances will be included to construct the logistic regression model for calculating propensity scores. Matching will be performed using nearest neighbor matching with a 1:1 ratio and a caliper value ranging from 0.01 and 0.1. As the secondary outcomes were exploratory, no adjustments were made for type I errors arising from multiple comparisons. The onset time of frailty will be analyzed using Kaplan-Meier survival curves and log-rank tests. Univariate Cox regression will be used to calculate hazard ratios (HR) and 95% CI. The safety outcomes will be analyzed using chi-square tests, continuity-corrected chi-square tests, or Fisher’s exact tests. Postoperative recovery quality scores, pain levels, sleep quality, grip strength, activities of daily living, and fall risk scores will be analyzed using generalized estimating equations.

For the sensitivity analysis of the primary outcome, different study populations and statistical method will be used to assess the robustness of the causal relationship between exposure factors and primary outcome. Subgroup analyses will be conducted for age, sex, ASA grade, smoking history, drinking history, marital status, and education level, with interaction effects being evaluated. All statistical tests will be two-sided, with *P* < 0.05 considered statistically significant.

### 2.11 Handling of missing data

Assuming the data are missing at random, missing data will be addressed using multiple imputation methods before conducting statistical analysis. The imputation will be performed five times with a maximum of 50 iterations. For continuous variables, the predictive mean matching model will be employed, whereas logistic regression models will be utilized for the imputation of categorical variables.

## 3 Discussion

As the aging population continues to expand, there is a heightened focus on frailty among elderly patients. Numerous studies have focused on the association between frailty and IC in community-dwelling older adults (Lee et al., 2014; Gutiérrez-Robledo et al., 2021; Chew et al., 2021; Shen et al., 2023), but such association in surgical patients—a highly vulnerable group—has been less explored. Postoperative frailty not only affects a patient’s recovery, disease outcomes, and quality of life, but it also significantly influences healthcare costs for families and society. This study has the potential to extend our means of predicting or identifying post-operative frailty while prompting clinical decision-makers to evaluate preoperative IC in elderly patients. Such assessments could facilitate the early detection of declines in IC, thereby enabling timely interventions post-surgery to optimize post-operative recovery in elderly patients and mitigate the risk of postoperative frailty.

Due to the limitations of objective conditions, we intend to assess patient outcomes via telephone follow-up, which leaves us with limited tools to evaluate frailty. The Frailty Index, an accurate tool developed by Rockwood et al. (2005), seems more appropriate for this study due to its deficit-emphasizing nature. However, our study relied solely on telephone follow-ups, making the application of the FI exceptionally difficult. Therefore, over some other 5-item scales, we chose the relatively complex Clinical Frailty Scale, which was also developed by Rockwood et al. (2005) and has been verified for its reliability. The CFS classifies frailty based on physical performance reported by patients. A lack of objective measures, such as grip strength and gait speed, may introduce information bias. And the CFS classification tree we used, while showing relatively high accuracy in scoring, is not entirely accurate and may still result in misclassification of frailty. Secondly, there are also some shortcomings in the measurement tools for IC. We used the five major domains recommended by the World Health Organization to quantify intrinsic capacity, and for each domain, we used the recommended tools. However, the existing IC assessment tools may fail to fully cover all the relevant dimensions of IC. Using a composite total score instead of a weighted score may also cause inaccuracy in the evaluation. In addition, considering the repeated measurements of outcome variables at multiple timepoints, multiple analyses may increase the likelihood of type I errors, particularly in secondary outcomes. Future studies with appropriate statistical corrections will be needed to confirm these findings.

## Footnotes

1
https://www.clinicaledc.com
